# Increasing the public health voice in global decision-making on nutrition labelling

**DOI:** 10.1186/s12992-019-0533-3

**Published:** 2020-01-03

**Authors:** Anne Marie Thow, Alexandra Jones, Carmen Huckel Schneider, Ronald Labonté

**Affiliations:** 1 Menzies Centre for Health Policy, School of Public Health, Charles Perkins Centre, The University of Sydney, Sydney, Australia; 2 The George Institute for Global Health, Sydney, Australia; 3 School of Epidemiology and Public Health, University of Ottawa, Ottawa, Canada

**Keywords:** Nutrition, Governance, Global standards, Front of pack labelling

## Abstract

To respond to the global noncommunicable disease (NCD) crisis, the Codex Alimentarius Commission (Codex), a multilateral United Nations body responsible for work on food standards, is developing global guidance for front of pack (FoP) nutrition labelling. Guidance from Codex regarding FoP nutrition labelling at the global level will almost certainly influence national policy making. This shift in Codex’s activities towards standards to address NCDs presents new risks for achievement of public health goals, as a result of the high level of industry involvement in this forum; there is a potential commercial conflict of interest held by manufacturers of products whose consumption could be discouraged by such guidance. In this Commentary, we examine the implications of Codex processes for developing robust global guidance on FoP nutrition labelling and identify opportunities to increase consideration of public health objectives. To date, there has been significantly higher representation of food industry compared to public health actors in Codex discussions on FoP nutrition labelling. Without a strong public health voice in Codex, the industry voice could dominate discussions on FoP nutrition labelling, such that subsequent global guidance prioritises future trade and profits over potential risks to public health. There is currently a critical window of opportunity for public health interests to be prioritised in this multisectoral international forum. The key public health priority for global guidance on FoP nutrition labelling is to ensure protection of policy space for national governments to implement strong and effective regulation, and allow scope for innovation. Public health actors can engage directly with Codex processes, at both the national and global level, and also need to raise awareness among domestic policy makers – including with Ministries of Agriculture and Industry, which often represent countries at Codex – regarding the importance and effectiveness of FoP labelling in NCD prevention. Increased engagement with Codex processes represents a tangible new opportunity to strengthen global governance for public health, and move towards improved coherence between trade policy and health protection goals.

## Introduction

Diet-related non-communicable diseases (NCDs) remain one of the leading causes of death and disability globally [1]. The World Health Organization (WHO) has repeatedly recommended front of pack (FoP) nutrition labelling as core to comprehensive policies to prevent NCDs, which must address food environments alongside behaviour change related policies [2, 3]. However, lack of specific guidance has been raised as a limitation to scaling up action globally. In particular, one of the concerns raised at the World Trade Organization (WTO) regarding FoP nutrition labelling initiatives was the lack of guidance from a recognized standards setting body on ‘best practice’ [4].

The lead global agency governing food labelling is not the WHO, however, but rather the Codex Alimentarius Commission (Codex), a multilateral United Nations (UN) body responsible for work on food standards. In 2017, Codex began working to establish global guidance for FoP nutrition labelling to inform global policy action. As a joint programme of WHO and the Food and Agriculture Organization of the United Nations (FAO), Codex’s work balances dual and potentially competing objectives of protecting consumer health and facilitating food trade. Codex guidance has in the past been highly influential for national nutrition labelling policy, with countries often adopting Codex guidance directly into national law [5]. This is evident, for example, in the widespread adoption of nutrient information panels globally.

Concerns have been raised about the institutional structure of Codex, and the extent to which it represents good global governance for public health – in particular around issues of access, transparency, representation and managing potential conflicts of interest [6]. The focus of Codex guidance historically has been on technical issues, primarily related to food safety and composition, which has legitimised industry involvement in developing guidance and standards. In addition to participation by Member States, Codex allows contributions by formal Observers. Observers contribute to decision making (although they cannot vote) and have access to draft documents. Over 60% of the Observers represent industry interests [7] many of them related to packaged and processed products, high in harmful fats, added sugars and sodium that are recognised as drivers of diet-related NCDs [8]. As a result, a shift in Codex’s activities towards regulations to address NCDs thus presents new risks associated with industry involvement, given the potential commercial conflict of interest held by manufacturers of products whose consumption could be discouraged by potential guidance. As a technical public health issue, it is also an area in which public health rather than industry expertise is most relevant. In this Commentary, we examine the Codex process for developing guidance on FoP nutrition labelling and highlight the urgency of increasing visibility and consideration of public health interests, in order to strengthen NCD prevention policy globally.

### Front of pack nutrition labels are core to NCD prevention

**Fig. 1:**
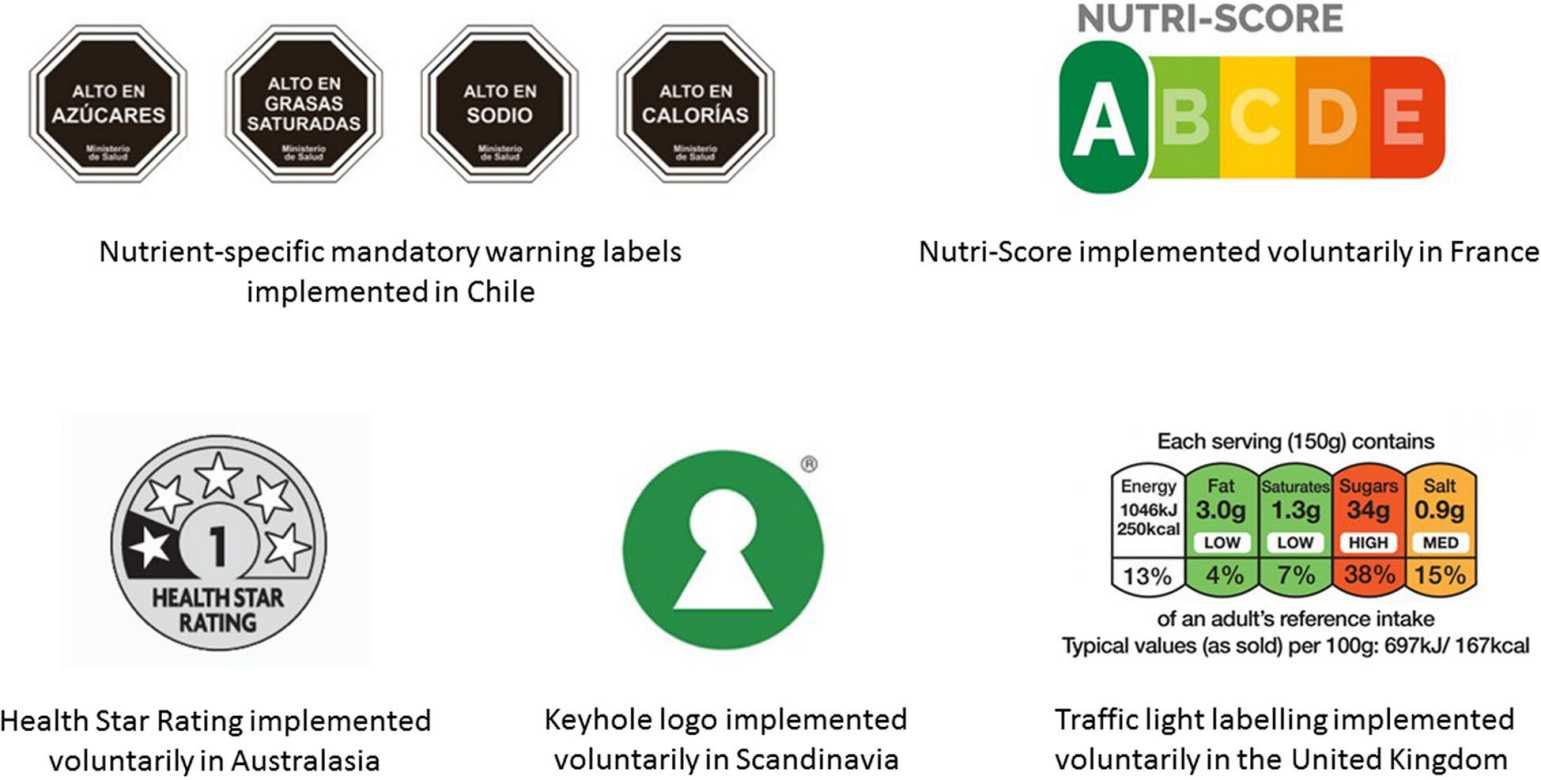
Examples of front of pack nutrition labels. Source: sample label images obtained from the government websites associated with each scheme

Over 30 governments have introduced requirements for symbols or other graphics as supplementary nutrition information on the main (front) panel of pre-packaged foods [9]. These include a range of interpretive and/or informative approaches, and positive and/or negative judgements (e.g. endorsement logos, warning labels [9], such as ‘Guideline Daily Amount labels’, warning labels, traffic light labels, and the ‘Health Star Rating’ system), which are being implemented under voluntary or mandatory arrangements (Fig. 1). Existing back of pack nutrient content labels are difficult for consumers to understand and interpret with respect to healthy food choices [9]. Evidence shows that addition of FoP nutrition labels aids consumer understanding of the nutritional quality of the product, encourages selection and purchase of healthier foods, and promotes reformulation of processed foods [10–13]. Although the evidence base continues to evolve, studies to date suggest that effective FoP nutrition labelling approaches should include mandatory labels that use interpretive elements, such as colours, symbols or words, underpinned by robust and transparent criteria for nutrient scoring or profiling [9]. There is global consensus that, in the longer term, FoP nutrition labels will contribute to comprehensive strategies to improve diets, and thereby reduce the risk of overweight, obesity and diet-related NCDs [3].

### Why does Codex guidance matter?

Guidance from Codex regarding FoP nutrition labelling at the global level will almost certainly influence national policy making. The Codex Alimentarius, a collection of internationally adopted food standards, guidelines and related texts, often becomes the basis for national policy and legislation [6]. Codex guidelines for ingredients lists and nutrient content presentation, presented on the back of pack, have been widely adopted globally as a mandatory requirement [14, 15]. This track record underpins Codex’s legitimacy and technical authority in developing guidance on FoP nutrition labelling.

Codex is recognised by the WTO as a standards setting body for food [16]. This means that Codex recommendations are used as a reference point for 1) interpreting Agreements of the WTO (particularly with respect to Technical Barriers to Trade and Sanitary and Phytosanitary Measures), and 2) informing arbitration of trade disputes related to food. National standards or policy measures that are ‘based on’ international standards have lesser requirements for justification in a trade context. For example, policies made in accordance with international standards are subject to a rebuttable presumption that they do not create unnecessary obstacles to international trade (see, for example, Articles 2.4 and 2.5 of the Agreement on Technical Barriers to Trade) [17].

When WTO member states exceed Codex standards, however, there are additional requirements for scientific justification under the Technical Barriers to Trade Agreement (under which these labelling measures fall) [18]. If these are not met, countries may face challenges from other WTO member states. Countries in which FoP labels are mandatory, including Chile, Thailand, Ecuador and Peru, have already been subject to discussions at the WTO about whether they are the ‘least trade restrictive alternative’ to achieving the policy goal of reducing diet-related NCDs [4]. While it is rare within the WTO system for matters to escalate to formal disputes, even threat of such challenges creates a barrier to policy innovation beyond Codex’s baseline, particularly in low-resource settings [6]. Such challenges can delay or weaken proposed public health regulations [19]. As a result, the decisions made in Codex regarding guidance on FoP nutrition labelling are likely to have a significant influence on national decision making on this issue.

### Influencing Codex decisions

As Codex’s role as a reference point in WTO discussions and disputes has increased, its rule-making processes have become more politicized [5, 6]. Codex’s members have grown rapidly, from the 44 who attended the inaugural meeting in 1962 to 189 members in 2018 (188 countries and the European Union), and there has been increased involvement of national trade officials. There has been increased attention and involvement of other intergovernmental organizations (IGOs), including the WTO Secretariat and regional trade bodies, which together now comprise 72 of 225 formal Observers (over 30%) [7]. In addition, non-state actors from the corporate sector and civil society have sought to play a greater role in the standard-setting process through direct involvement as Observers; 140 of the formal Observers at Codex represent industry interests (63%, including some IGOs with marketing/commodity mandates), and 13 represent public interests (10%). Industry and other non-government actors also sometimes form part of member state delegations, and engage in less visible efforts at a national level to influence the negotiating positions of state actors [6].

To date, there has been significantly higher representation of food industry compared to public health actors in Codex discussions on FoP nutrition labelling [20]. The electronic Working Group (eWG) established on this issue in 2017 included 44 Member Countries and 20 Observers, including 14 non-governmental organizations representing the food industry,^1^ notably the sugar and beverages sectors, and only four representing consumer and public health interests (Consumers International, the International Association of Consumer Food Organizations, the World Federation of Public Health Associations and World Obesity). At the Committee on Food Labelling meeting in May 2019, at which FoP nutrition labelling was discussed, there were 19 industry Observers making oral submissions to negotiations, and only seven public interest Observers [21].

Without a strong public health voice in Codex, the industry voice could dominate discussions on FoP nutrition labelling, such that subsequent global guidance prioritises future trade and profits over potential risks to public health. A core function of Codex “is to guide and promote the elaboration and establishment of definitions and requirements for food, to assist in their *harmonization* and, in doing so, *facilitate international trade*” [emphasis added] [22]. From this perspective, the diversity of labelling approaches appearing globally is framed as a problematic barrier to trade, rather than an evolution of the public health evidence base [23].

The eWG developed an agenda paper and draft guidance for further discussion in 2019 [24]. Notably, this draft does not refer to NCDs, highlighting the critical need for improved policy coherence. Tensions between public health interests in reducing consumption of foods associated with NCD risk and industry objectives to promote trade and consumption are evident. For example, some of the proposals raised in Codex discussions on FoP nutrition labelling seem to reflect industry concerns about innovative ‘strong’ FoP nutrition labels such as Chile’s ‘stop sign’ warnings, which signal product unhealthfulness and discourage consumption. These include a proposal for only one FoP nutrition label to be recommended in each country or region, and debates about whether nutrient-specific warning style labels are included within the definition of FoP labelling and thus the guideline’s remit. These issues potentially jeopardize progression of innovative policies currently being developed in Argentina, Brazil, Canada, Guatemala, India, Portugal, South Africa, and Spain.

The draft guidance also contains process requirements, including whether FoP nutrition labelling should be government-led, and whether governments should be required to involve industry and other stakeholders in developing national policy. From a public health perspective, it is essential that Codex guidance clearly demarcate where there is potential for commercial conflicts of interest to interfere with the achievement of legitimate health objectives in policymaking, and ensure that governments retain sufficient policy space and flexibility to manage them. This is particularly important in the light of provisions giving industry actors access to decision makers at the national level in recent trade agreements. For example, provisions regarding regulatory coherence in the Korea-United States (KORUS) Free Trade Agreement included commitments to allow persons (including corporate representatives) from the other party to participate in policy decisions that relate to trade [18]. This same provision is now part of the Comprehensive and Progressive Trans-Pacific Partnership agreement.

Draft guidance was discussed at the Codex Committee on Food Labelling (CCFL) in May 2019. A similar imbalance in representation was seen in these discussions as in the eWG, with seven public interest and 19 industry Observer delegations present. Industry also participated on country delegations; there were nine individuals from the Coca-Cola Corporation present, including five dotted throughout the country delegations of Australia, Mexico, India, Argentina and Chile [24]. Discussion included opposition by some delegates to innovative labelling approaches such as Chile’s ‘high in’ stop signs. The matter was stood over for further consideration by the eWG and a further meeting of CCFL in 18 months’ time, which means this is an opportune time to raise the public health voice in Codex discussions on labelling, to ensure a positive outcome for public health [24].

## Conclusion: raising the public health voice

A visible and compelling public health voice in the global governance of FoP nutrition labelling will require communicating *public health priorities* to the right *institutions* and *actors*.

The key public health priority for global guidance on FoP nutrition labelling is to ensure protection of policy space for national governments to implement strong and effective regulation. In particular, it is critical that guidance clearly allows for national policies that pursue legitimate public health nutrition objectives, by allowing room for 1) diversity and innovation to respond to the needs of their populations and food system context, 2) strong (including mandatory) regulation, and 3) governments to base approaches on both international and national evidence, as appropriate. Given the emerging nature of the evidence base, and the limited resources of some governments, the guideline also needs to allow for policy development based on evidence of *potential* risk, for protection of human health. Any guidance developed by Codex also needs to allow governments policy space to prevent and manage potential conflicts of interest in developing national FoP nutrition labelling schemes [25]. For example, in order to limit industry influence on policy design, governments may elect to engage with industry during implementation, but limit engagement during policy development in order to protect achievement of public health nutrition goals.

Presenting the evidence in the right institutional context requires an understanding of the global governance of labelling. The key institution within which to increase the participation of actors representing public health interests is Codex itself. Public health actors can engage with Codex processes, at both the national and global level (Box 1). Nutrition labelling is also within the institutional remit of the WHO and WTO, both of which have formal links to Codex. As a result, formal recognition within Codex guidance of concurrent WHO activities and recommendations regarding FoP nutrition labelling will be vital for promoting positive public health outcomes and policy coherence. Explicit reference to WHO’s program of work on FoP nutrition labelling will also make it easier for Codex guidance to be updated in response to WHO evidence reviews.

**Table:** 

**Box 1: Engaging with Codex**	
Public health actors can help to bring public health concerns to the attention of Codex decision makers through:	
1) engaging with their national Codex Contact Point, for example, signing up to receive notifications and making submissions to them;	
2) becoming a member of the national delegation to Codex meetings;	
3) becoming an Observer (as an international organization); and/or.	
4) joining an existing Observer organization and participating in submissions to Codex processes.	
For more information, please visit https://blogs.plos.org/globalhealth/2018/06/cracking-the-codex/	

At the country level, public health actors need to raise awareness among policy makers of the importance and effectiveness of FoP labelling in NCD prevention. In this context, it’s highly relevant that Codex Contact Points are often situated within Ministries of Agriculture, Trade and/or Industry and may have little awareness of the public health implications of the current Codex discussions on labelling (or, indeed, of discussions within Ministries of Health). Increased awareness of the governance of nutrition labelling across multiple sectors, and the various requirements for evidence – for example, in trade forums – will also be important for nutrition labelling researchers. There is a clear need for policy-relevant (and policy aware) multidisciplinary research on FoP labelling to inform design of effective policies.

As has been argued for some time, strengthening global health governance will require addressing ongoing incoherence between trade policy and health protection goals. Fulsome public health participation around FoP labelling at both national and international levels offers a new engagement site to do so.

## Data Availability

Not applicable.

## Abbreviations

CCFL: Codex Committee on Food Labelling
Codex: Codex Alimentarius Commission
eWG: Electronic Working Group
FAO: Food and Agriculture Organization of the United Nations
FoP: Front of pack
IGOSs: Intergovernmental organizations
KORUS: Korea-United States
NCDs: Non-communicable diseases
UN: United Nations
WHO: World Health Organization
WTO: World Trade Organization

## Footnotes

1 Asociación Latinoamericana de Avicultura (ALA), CEFS - Comité Européen des Fabricants de Sucre, Food Drink Europe, Food Industry Asia, Institute of Food Technologists®, International Chewing Gum Association, International Council on Amino Acid Science, International Council of Beverages Associations, International Council of Grocery Manufacturers Association, International Dairy Federation, International Fruit and Vegetable Juice Association, International Organization of Vine and Wine, International Special Dietary Foods Industries, World Sugar Research Organization)
